# Health Related Quality of Life in Patients with Bladder Cancer Receiving a Radical Cystectomy

**DOI:** 10.3390/cancers15245830

**Published:** 2023-12-13

**Authors:** Riccardo Mastroianni, Andrea Iannuzzi, Alberto Ragusa, Gabriele Tuderti, Mariaconsiglia Ferriero, Umberto Anceschi, Alfredo Maria Bove, Aldo Brassetti, Leonardo Misuraca, Simone D’Annunzio, Salvatore Guaglianone, Rocco Papalia, Giuseppe Simone

**Affiliations:** 1 Department of Urology, IRCCS “Regina Elena” National Cancer Institute, 00144 Rome, Italy; gabriele.tuderti@ifo.it (G.T.); maria.ferriero@ifo.it (M.F.); umberto.anceschi@ifo.it (U.A.); alfredo.bove@ifo.it (A.M.B.); aldo.brassetti@ifo.it (A.B.); leonardo.misuraca@ifo.it (L.M.); simone.dannunzio@ifo.it (S.D.); salvatore.guaglianone@ifo.it (S.G.); giuseppe.simone@ifo.it (G.S.); 2 Department of Urology, Fondazione Policlinico Universitario Campus Bio-Medico, 00128 Rome, Italy; andrea.iannuzzi@unicampus.it (A.I.); alberto.ragusa@unicampus.it (A.R.); rocco.papalia@policlinicocampus.it (R.P.)

**Keywords:** bladder cancer, radical cystectomy, Health-Related Quality of Life, robotic surgery, minimally invasive surgery, open radical cystectomy

## Abstract

**Simple Summary:**

Health-related quality of life (HRQoL) in patients with bladder cancer receiving radical cystectomy is too often overlooked, with the scientific community being mostly focused on survival outcomes and postoperative complications, which are always considered the main outcomes of any genitourinary cancer treatment. This study aimed to identify the most impaired HRQoL features in patients receiving a radical cystectomy, compared to a healthy population control, as well as patients’ recovery after surgery, providing high-level evidence for HRQoL assessment, in order to minimize the undeniable impact of surgery on daily life.

**Abstract:**

Radical Cystectomy (RC) and Urinary Diversion (UD) is a complex surgery associated with a significant impact on health-related quality of life (HRQoL). However, HRQoL assessment is too often overlooked, with survival and complications being the most commonly investigated outcomes. This study aimed to identify the most impaired HRQoL features in patients receiving RC, compared to a healthy population (HP) control, as well as patients’ recovery after surgery, differentiating between patients receiving ORC and RARC. Patients with Bca, who were candidates for RC with curative intent, were enrolled in the “BCa cohort”. HRQoL outcomes were collected with an EORTC QLQ-C30 questionnaire. These were collected at baseline, and then at 6-, 12- and 24 mo after surgery in the BCa cohorts, and at baseline in the HP cohort. A 1:1 propensity score matched (PSM)-analysis, adjusted for age, Charlson Comorbidity Index (CCI) and smoking history, was performed. Between January 2018 and February 2023, a total of 418 patients were enrolled in the study, 116 and 302 in the BCa and HP cohorts, respectively. After applying the 1:1 propensity scored match (PSM) analysis, two homogeneous cohorts were selected, including 85 patients in each group. Baseline HRQoL assessment showed a significant impairment in terms of emotional and cognitive functioning, appetite loss and financial difficulties for the BCa cohort. Among secondary outcomes, we investigated patients’ recovery after RC and UD, comparing HRQoL outcome questionnaires between the HP and BCa cohorts at 6-, 12- and 24 mo after surgery, and a subgroup analysis was performed differentiating between patients receiving ORC and RARC with totally intracorporeal UD. Interestingly, ORC compared to RARC provided a major impact on HRQoL recovery across the early, mid and long term. In particular, the ORC cohort experienced a major impairment in terms of symptoms scales items such as fatigue, nausea and vomiting, pain and appetite loss. Consequently, comparing ORC and RARC vs. HP reported a major HRQoL impairment in the ORC cohort, possibly defining a benefit of RARC in early, mid- and long-term recovery. To conclude, this study confirmed the undeniable impact of RC on HRQoL. Interestingly, we highlighted the benefit of RARC in early, mid- and long-term recovery, expressed as less impairment of symptoms scales.

## 1. Introduction

The Health-Related Quality of Life (HRQoL) of patients receiving surgical treatments for genitourinary cancers are still too often overlooked in the current literature, which is predominantly focused on oncologic and postoperative outcomes. However, HRQoL is part of patients’ wellbeing; therefore, more efforts are needed to extensively describe and evaluate the impact of surgery on HRQoL [1,2,3]. To date, Bladder Cancer (BCa) is the 10th most diagnosed cancer in the general population and approximately 25% of patients also have a diagnosis of a Muscle Invasive (MI) disease. The gold-standard treatment for non-metastatic MIBC and for recurrent BCG failure in high-grade (HG) non-muscle invasive BC (NMIBC) is Radical Cystectomy (RC) with pelvic lymph-node (LN) dissection and urinary diversion (UD) [4]. This major surgical procedure has a noticeable impact on HRQoL. To date, Open RC (ORC) is still considered the reference option for the surgical management of BC, although the robotic approach has gained popularity due to its potential benefits in terms of a lower morbidity, and particularly for lower perioperative blood loss and transfusions [5]. The available randomized controlled trials aimed at comparing ORC and RARC with extracorporeal UD showed comparable oncological outcomes and no significant differences in terms of peri- and post-operative complication rates between surgical approaches [6,7,8,9]. Recently, results from an RCT aimed at comparing ORC and RARC with totally intracorporeal UD provided a 50% overall transfusion rate reduction in the RARC cohort [10]. However, RC is still considered a complex surgery associated with a mortality rate of 2.1–3.2% at 30 days and 3.4–8.0% at 90 days. At the same time, RC is burdened by high rates of complications, which range from 50–88% for low-grade Clavien complications and from 30–42% for high-grade Clavien complications (Clavien ≥ 3), while long-term complications are mostly related to UD performed [5]. As a result, the overall impact of RC on HRQoL is widely recognized, and it is strongly related to the high morbidity following the surgical procedure and the major impact of UD on daily life activities [11]. In particular, the most significant impact of RC on HRQoL seems to be reported in terms of physical and social functioning. RARC is constantly increasing due to potential advantages in terms of faster recovery and minor impairment on HRQoL. However, the impact of this surgical approach on HRQoL is still underestimated [12]. There is still lack of valuable data reporting HRQoL outcomes in this specific setting. Therefore, more efforts are needed to extensively evaluate the HRQoL of patients receiving RC.

In this study, we aimed to compare HRQoL between BCa patients receiving RC versus the general healthy population in order to identify the mostly impaired HRQoL features of patients who undergo surgical management of BCa.

## 2. Material and Methods

### 2.1. Study Design

Inclusion criteria:

- cT2-4/N0/M0 or BCG failure and high-grade non-muscle-invasive urothelial carcinoma;
- patients able to provide a written informed consent and compliant with study protocol time schedule.

Exclusion criteria:

- anesthesiologic contraindications to RARC [severe cardiovascular disease, early cerebrovascular disease (within 30 d), retinal vascular disease and ventriculoperitoneal shunt];
- palliative surgery.

Patients with BCa who were candidates for cystectomy with curative intent, and whose data were collected in a prospective Randomized Controlled Trial (Clinical Trials: NCT03434132), were enrolled in the “BCa cohort”. Clinical and HRQoL data from General Practitioner outpatients were collected, and patients were enrolled in the “healthy population (HP) cohort”. BCa cohort patients received questionnaires following signature of informed consent for RC and before-surgery inclusion criteria were age ≥ 18 years old and no previous diagnosis of BCa or any genitourinary cancer disease. Institutional board approval was obtained, and patients were included in accordance with the principle of confidentiality. Informed consent was obtained from all subjects involved in the study.

### 2.2. Procedures

RC was performed by a surgical team with established experience for both ORC and RARC (more than 50 procedures per year, in the last 2 years prior to enrolment). Concerning RARC, the UD was performed totally intracorporeal in all cases. Both procedures have been previously described. After discharge, BCa patients were followed-up monthly for the first 3 months, and then at 6 mo intervals after surgery. Abdominal, pelvic and chest CT-scan were performed at baseline and at 6 mo follow-up, and then once a year.

### 2.3. Outcomes

HRQoL outcomes were collected by the European Organization for Research and Treatment of Cancer (EORTC), and the generic (QLQ-C30) questionnaire. These were collected at baseline, and then at 6-, 12- and 24 mo after surgery in BCa cohorts, and at baseline in HP cohort.

The QLQ-C30 consists of a combination of multi-item scales and single-item measures, encompassing a comprehensive assessment of various aspects. It includes five separate functional categories (physical, role, emotional, cognitive and social functioning) as well as three symptom categories (fatigue, nausea and vomiting, pain). Furthermore, it assesses overall health status and quality of life, while also incorporating six individual items to measure breathlessness, sleep problems, reduced appetite, bowel issues, diarrhea and financial challenges. Each group of multiple items within a category consists of unique questions, with no repetition in questions across different categories. Scores for all scales and single-item measures range from 0 to 100. In this scoring system, a higher value signifies a more pronounced response. Consequently, elevated scores within the functional scales reflect elevated or healthier levels of functioning. Similarly, a heightened score in the global health status/QoL scale corresponds to an enhanced quality of life. Conversely, a higher score within a symptom scale or item is indicative of a greater degree of symptomatology or issues. It is important to note that all multi-item scales share the same scoring range of 0 to 100, with the exception of sexual functioning. Elevated scores across most multi-item scales, excluding sexual functioning, suggest an elevated level of symptomatology or challenges. Conversely, a higher score in the sexual functioning scale indicates an elevated level of functioning in that specific domain [13,14].

The primary endpoint was to evaluate which HRQoL features were mostly impaired in the BCa cohort compared to the general HP. Among secondary outcomes we investigated patients’ recovery after RC and UD, comparing HRQoL outcome questionnaires between HP and BCa cohorts at 6-, 12- and 24 mo after surgery, further reporting differences between ORC and RARC patients.

### 2.4. Statistical Analysis

The descriptive statistics for categorical variables primarily emphasized frequencies and proportions. For continuously coded variables, we presented their means. We utilized Student *t*-tests to compare continuous variables and Fisher’s exact and Chi-Square tests to compare categorical variables.

Due to inherent disparities between patients undergoing RC and the healthy population, we conducted a 1:1 propensity score matched (PSM) analysis with a caliper of 0.1 to account for these differences. Employing the propensity score method helped mitigate the common biases associated with conventional multivariable modeling. We adjusted for age, Charlson Comorbidity Index (CCI), and smoking history as variables. Paired *t*-tests were used to compare normally distributed continuous HRQoL variables. The significance threshold was set at *p* < 0.05. The statistical analysis was performed using the Statistical Package for Social Science (SPSS, IBM, version 22.0, Armonk, NY, USA).

## 3. Results

Between January 2018 and February 2023, a total of 418 patients were enrolled in the study, with 116 and 302 in the BCa and HP cohorts, respectively. Baseline features were reported (Table 1). Statistically significant differences were detected between the two groups concerning age (*p* = 0.006), smoking history (*p* < 0.001), diabetes (*p* = 0.034) and CCI (*p* < 0.001). After applying 1:1 PSM analysis, two homogeneous cohorts were selected, including 85 patients for each group (Table 2). Analysis of the HRQoL outcomes in the PSM cohorts showed statistically significant differences in terms of emotional (*p* = 0.011) and cognitive functioning (*p* = 0.028), appetite loss (*p* = 0.011) and financial difficulties (*p* = 0.018) (Table 3).

**Figure 1:**
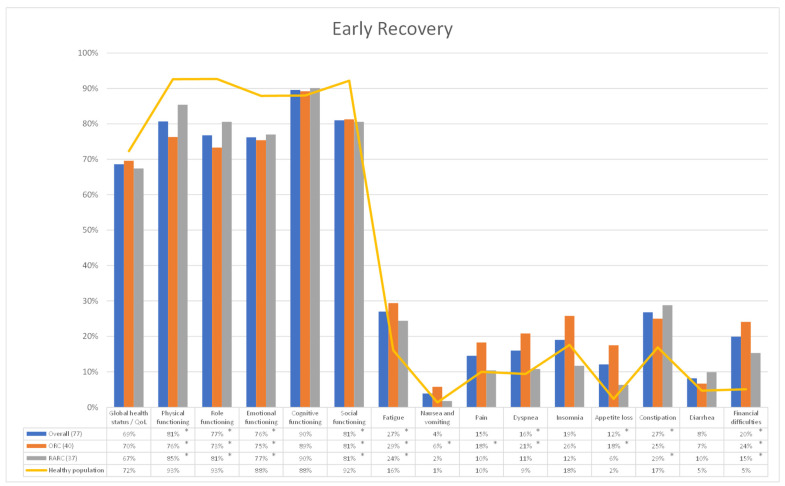
Comparison of HRQol outcomes between healthy population and Bladder Cancer (BCa) cohort 6 mo after Radical Cystectomy. Overall, entire cohort of patients underwent RC. ORC: Open Radical Cystectomy cohort. RARC: Robot-assisted Radical Cystectomy cohort. Overall, ORC and RARC cohorts were compared to healthy population cohort using Student *t* test. * all *p* < 0.05.

**Figure 2:**
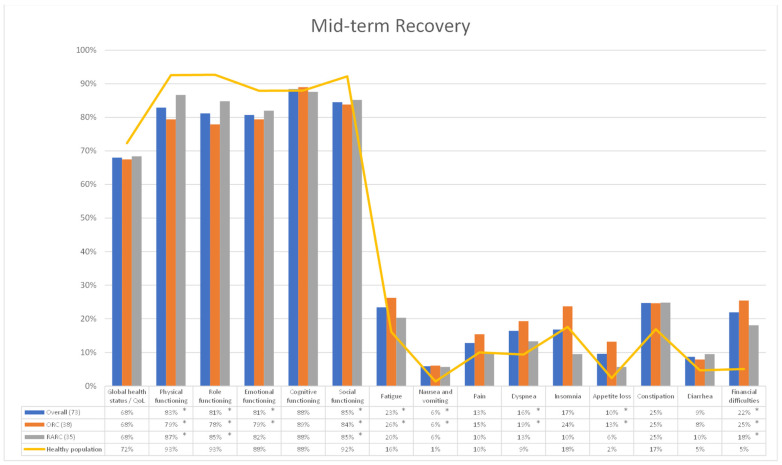
Comparison of HRQoL outcomes between healthy population and Bladder Cancer (BCa) cohort 12 mo after Radical Cystectomy. Overall, entire cohort of patients underwent RC. ORC: Open Radical Cystectomy cohort. RARC: Robot-assisted Radical Cystectomy cohort. Overall, ORC and RARC cohorts were compared to healthy population cohort using Student *t* test. * all *p* < 0.05.

**Figure 3:**
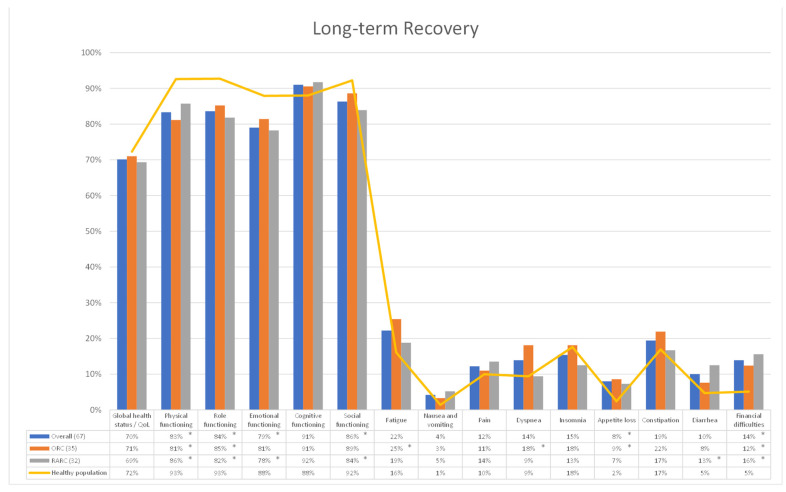
Comparison of HRQol outcomes between healthy population and Bladder Cancer (BCa) cohort 2 mo after Radical Cystectomy. Overall, entire cohort of patients underwent RC ORC: Open Radical Cystectomy cohort RARC: Robot-assisted Radical Cystectomy cohort. Overall, ORC and RARC cohorts were compared to healthy population cohort using Student *t* test. * all *p* < 0.05.

Among secondary outcomes we investigated patients’ recovery after RC and UD, comparing HRQoL outcome questionnaires between the HP and BCa cohorts at 6-, 12- and 24 mo after surgery, and a subgroup analysis was performed differentiating patients receiving ORC and RARC with totally intracorporeal UD. A total of 77 (ORC: 40—RARC: 37), 73 (ORC: 38—RARC: 35) and 67 (ORC: 35—RARC: 32) questionnaires were collected from patients who underwent RC 6-, 12- and 24-mo after surgery, respectively. At early recovery (6-mo of follow-up), all patients receiving RC, both the ORC and RARC cohorts, experienced a significant impairment, compared to the HP, in terms of *physical*, *role*, *emotional* and *social functioning* (all *p* < 0.001), as well as *fatigue* (*p* < 0.024) and *financial difficulties* (*p* < 0.003). In particular, the ORC cohort showed a statistically significant deterioration in terms of *nausea and vomiting* (*p* = 0.043), *pain* (*p* = 0.027), *dyspnea* (*p* = 0.004) and *appetite loss* (*p* < 0.001); while the RARC cohort reported a major impairment in terms of *constipation* (*p* = 0.025) (Figure 1). At mid-term recovery (12-mo of follow-up), all patients receiving RC, both the ORC and RARC cohorts, experienced a significant impairment in terms of *physical* (*p* < 0.040), *role* (*p* < 0.037) and *social functioning* (*p* < 0.045) and *financial difficulties* (*p* < 0.001), while only the ORC cohort showed a higher deterioration in *emotional functioning* (*p* = 0.030), *fatigue* (*p* = 0.009), *nausea and vomiting* (*p* = 0.023), *dyspnea* (*p* = 0.012) and *appetite loss* (*p* = 0.001) (Figure 2). Finally, at long-term recovery (24 mo follow-up), all patients receiving RC, regardless from surgical approach, experienced a significant impairment in terms of *physical* (*p* < 0.045) and *role functioning* (*p* < 0.039) and *financial difficulties* (*p* < 0.031). On the one hand, the ORC cohort, compared to the HP group, showed a higher level of *fatigue* (*p* = 0.022), *dyspnea* (*p* = 0.028) and *appetite loss* (*p* = 0.035). On the other hand, the RARC cohort provided higher impairment in terms of *social functioning* (*p* = 0.022) and *diarrhea* (*p* = 0.040) (Figure 3).

## 4. Discussion

RC is still considered a complex surgery associated with higher morbidity [5,15]. Indeed, the scientific community has predominantly focused its efforts on survival outcomes and perioperative complications, which are always considered the major goals in any genitourinary cancer treatment [16,17]. Moreover, since the widespread diffusion of minimally invasive surgery, a comparison between different surgical approaches has been considered of primary importance [18]. It is clear that, the increasing popularity of the robotic approach is primarily related to the awaited advantages it offers, particularly in terms of reducing morbidity and improving Health-Related Quality of Life (HRQoL). Despite this, the impact of robotic surgery on HRQoL has often been overlooked [19]. However, the spread of robotic surgery in the field of RC is still limited, being the prerogative of tertiary referral centers. The primary obstacle to a widespread adoption of Robot-Assisted Radical Cystectomy (RARC) in clinical practice was the longer operative time resulting from the inherent complexity of performing a completely intracorporeal urinary diversion (i-UD). Although the last Pasadena Consensus Panel [20] reported benchmarks for RARC, emphasizing the significance of attempting an i-UD in all cases, the extracorporeal approach continues to be the preferred approach in practice following RARC. Nowadays, all the existing randomized controlled trials (RCTs) [6,7,8,9] were distinguished by an extracorporeal approach in performing UD, strongly undermining the potential benefits of a totally minimally invasive procedure the expected advantages that a completely minimally invasive procedure could offer. In prior studies, Gill et al. [21] highlighted limitations in available RCTs, noting non-comparable surgical experience between RARC and ORC, resulting in a low patient accrual rate (only 25% of eligible patients). Most contemporary studies comparing ORC and RARC have consistently demonstrated RARC’s advantages in terms of minor blood loss, at the cost of longer operative times [22]. However, minor variations between trials in terms of all other perioperative outcomes have been reported [23]. Nix et al. [6] described benefits of RARC in terms of lower estimated blood loss (EBL) (273 mL vs. 564 mL; *p* = 0.0003), faster time to bowel movement (3.2 d vs. 4.3 d; *p* = 0.0033) and lower in-house analgesia (93.6 mg vs. 151.6 mg; *p* = 0.0011), despite longer OT (4.2 h vs. 3.5 h; *p* < 0.0001). Similarly, Bochner et al. [7] emphasized RARC’s benefits in terms of EBL (500 mL vs. 681 mL; *p* = 0.012), while reporting longer OT (RARC 464 min vs. ORC 330 min; *p* < 0.001) and comparable hospital stay (HS) (8 d vs. 8 d; *p* = 0.9). In contrast, the CORAL study [8] did not conclusively support the benefit of RARC in terms of EBL (585 mL vs. 808 mL; *p* = 0.070), although a trend towards significance was described. However, they reported comparable HSs (11.9 d vs. 14.4 d; *p* = 0.3) and longer OT in RARC (389 min vs. 293; *p* < 0.001), with a slight difference in terms of time to oral solids (4.0 d vs. 7.5 d; *p* = 0.049). Finally, the RAZOR trial [9] highlighted RARC benefits such as reduced EBL (300 mL vs. 700 mL; *p* < 0.0001) and lower intra- (13% vs. 34%; *p* < 0.0001) and post-operative (25% vs. 40%; *p* = 0.0089) transfusion rates. Consistent with these findings, the first RCT comparing ORC vs. RARC with totally i-UD confirmed significantly lower transfusions rates in the RARC cohort (22% vs. 41%; *p* = 0.046), with comparable intra-operative (5% vs. 10%; *p* = 0.490) and a trend towards significantly lower post-operative (22% vs. 40%; *p* = 0.070) transfusions rates. Interestingly, among functional outcomes, we described comparable day-time continence outcomes between ORC and RARC with intracorporeal orthotopic neobladder both in terms of continence recovery probabilities and through quantitative assessments. Specifically, 61% and 59% of patients achieved complete dryness in the RARC and ORC cohorts, respectively, providing a good continence status also in robotic cohort. However, while comparable night-time continence recovery probabilities were described, a better night-time quantitative analysis of pad use and wetness were reported for ORC cohort [24].

Overall, the impact of surgery on HRQoL should be emphasized alongside survival and QoL, being two faces of the same moon [25]. To date, HRQoL outcomes following RC are underestimated. In particular, BCa patients seem to experience the worst HRQoL compared to patients with other pelvic cancers [26]. Indeed, HRQoL is undoubtedly one of the most challenging outcomes to analyze, involving the personal and subjective sphere of each individual patient. To solve this issue several validated questionnaires were developed [27].

The primary endpoint of this study was to compare HRQoL between BCa patients receiving RC versus a general HP, in order to identify the most impaired HRQoL features in patients who undergo surgical management of BCa (ORC and RARC). After a 1:1 PSM analysis, *emotional functioning* and *cognitive functioning* were the most impaired functional scales items in patients with BCa receiving RC. This result is pretty easy to recognize. A new diagnosis of BCa requiring RC certainly played a major impact in the emotional aspect of each patient, due to unawareness of life expectancy, as well as the known possible severe complications that a major surgical treatment such RC could provide. As a result, *appetite loss* is one of the main symptoms and scaled items suffered by patients. To face these aspects, preoperative counselling should be stressed, with the aim of raising awareness of BCa disease and its surgical treatment, trying to reduce the emotional impairment of patients, but nevertheless recognizing the unavoidable emotional impact of a BCa diagnose requiring RC on patient perspectives. Interestingly, BCa patients experienced a major impairment in *financial difficulties*, which was probably related to the costs incurred by patients during diagnostic management, as well as days of work lost. This is a major unmet need that welfare should face in order to minimize individual costs that each patient has to afford.

As already pointed out, minimally invasive surgery has gained popularity, particularly due to potential benefits in terms of lower morbidity. In the field of RC, robotic surgery provided comparable oncologic outcomes and similar perioperative complication rates [6,7,8,9]. Recently, a direct relationship between surgical outcomes and the subjective evaluation of HRQoL has been demonstrated. Interestingly, the results we obtained recognized 1 yr USC pentafecta and trifecta achievement as predictors of a 2 yrs unmodified global evaluation of HRQoL [28]. Therefore, improving surgical quality has been recognized the primary goal for reducing impact of RC on HRQoL, further confirming that QoL and survival are two faces of the same moon. However, if the impact of RC on HRQoL has always been overlooked, only a limited number of studies have examined this particular matter, directly comparing RARC and ORC [19,29]. To date, all available RCTs comparing ORC vs. RARC reported no significant differences in terms of HRQoL outcomes. The RAZOR trial described no significant differences between RARC and ORC when it came to all the endpoints measured by the Functional Assessment of Cancer Therapy-Vanderbilt Cystectomy Index (FACT-VCI). In particular, both groups experienced a significantly higher emotional wellbeing score at 3 and 6 months than at baseline [9]. Moreover, both groups had significant improvements in mean total FACT-VCI score 6 months after surgery compared to baseline. Meanwhile, the CORAL trial briefly examined Health-Related Quality of Life (HRQoL) comparisons between minimally invasive (robotic and laparoscopic) approaches and ORC. Their findings indicated no statistically significant differences between the groups at any measured endpoint [6]. Similarly, Bochner et al. [5] did not observe any disparities between the RARC and ORC groups regarding HRQoL changes from baseline to 3 and 6 months when analyzing various items from the EORTC QLQ-C30 questionnaire.

Messer et al. also reported no significant distinctions in FACT-VCI scores at 3-, 6-, 9- and 12-months post-surgery [11]. The only exception was a slightly lower score in physical well-being at 6 months in the ORC group (HR 2.5, 95%CI 4.8 0.23; *p* = 0.04). However, the clinical relevance of this 2.5 point difference is considered to be minimal. In all other aspects, no impact of the surgical approach on HRQoL scores was evident at any of the assessed time points. This absence of differences persisted across all other domains and time points evaluated [9]. Recently, we established comparable outcomes between RARC and ORC with regard to all HRQoL domains, barring minor aspects such as *insomnia*, *abdominal bloating and flatulence*. Afterwards, with an analysis of early outcomes (6 mo of follow-up), we reaffirmed the noticeable influence of RC on self-assessed HRQoL outcomes. Both the ORC and RARC cohorts underwent a substantial deterioration in *physical*, *role* and *sexual functioning*, accompanied by a significant impact on *body image*, *abdominal bloating and flatulence*, *fatigue*, and *constipation*. While patients who underwent ORC demonstrated a greater likelihood of experiencing a considerable decline in HRQoL, particularly evident in a more pronounced impairment of symptoms scale, such as *dyspnea*, *appetite loss*, *diarrhea* and *urinary symptoms and problems*, the RARC group exhibited an unexpectedly significant decline in terms of future perspective. However, no discernible distinction emerged between the two groups when assessing changes over time, underscoring the marginal role of the surgical approach in influencing HRQoL outcomes, even in the early stages of postoperative recovery. Interestingly, narrowing down the 1 yr HRQoL outcomes analysis to patients who underwent PIB, we confirmed the significant impact of surgery on HRQoL; even when analyzing items across time and between cohorts we reported the overall positive impact of RARC on aspects such as *body image* and *sexual functioning*, underlining the expected advantages of a completely minimally invasive approach in improving HRQoL [24].

Among the secondary outcomes we investigated in this study, patients’ recovery after RC and UD, comparing the HRQoL outcome questionnaires between the HP and BCa cohorts at 6-, 12- and 24 mo after surgery, and a subgroup analysis was performed differentiating patients receiving ORC and RARC with totally intracorporeal UD. Interestingly, ORC compared to RARC provided a major impact on HRQoL recovery for the early, mid and long term. In particular, the ORC cohort experienced a major impairment in terms of symptoms and scaled items such as *fatigue*, *nausea and vomiting*, *pain* and *appetite loss.* Consequently, comparing ORC and RARC vs. HP revealed a major HRQoL impairment in the ORC cohort, possibly defining a benefit of RARC in early, mid- and long-term recovery. However, it must be considered that self-reported HRQoL has always been challenging to globally evaluate and interpret. Therefore, further analysis is needed to extensively evaluate differences between surgical approaches.

The present study is not devoid of limitations. First, RC was performed in a single high-volume referral center; therefore, the results obtained may not be widely generalizable. There is also the absence of a longitudinal assessment of HRQoL outcomes in the HP cohort. Moreover, the small sample size due to the need to perform PSM analysis. Finally, the higher rates of orthotopic neobladder in BCa patients, than those reported in general practice, could jeopardize HRQoL outcomes and we acknowledge the potential pitfalls of self-assessed EORTC questionnaires to objectively evaluate patients’ HRQoL.

## 5. Conclusions

This study confirmed the undeniable impact of RC on HRQoL, particularly expressed as a decline in *emotional* and *cognitive functioning* with a consequent impact on *appetite loss*. Moreover, BCa patients experienced a significant impact on *financial difficulties*, as a possible consequence of costs incurred by patients during the diagnostic management, as well as days of work lost. Interestingly, this study highlighted the benefit of RARC in early, mid- and long-term recovery, expressed as less impairment of symptom scales such as *fatigue*, *nausea and vomiting*, *pain* and *appetite loss.*

## Figures and Tables

**Table 1:** Baseline features of the entire cohort.

**Variables** **N (%)** **Mean (SD)**	**Overall** **(418)**	**Bladder Cancer** **(116)**	**Healthy Population** **(302)**	***p*** **Value**
Gender				0.08
Male	278 (67)	85 (73)	193 (64)	
Female	140 (33)	31 (27)	109 (36)	
Age (yrs)	61 (11.6)	63 (9.4)	59 (12.2)	0.006
BMI (kg/m^2^)	26 (3.8)	26 (3.7)	26 (3.9)	0.78
Smoking history				<0.001
Current	95 (23)	42 (36)	53 (17)	
Former	106 (25)	50 (43)	56 (19)	
Never	217 (52)	24 (21)	193 (64)	
Diabetes	37 (9)	16 (14)	21 (7)	0.034
Hypertension	164 (39)	53 (46)	111 (37)	0.19
Myocardial infarction history	43 (10)	11 (10)	32 (11)	0.86
Charlson Comorbidity Index	3 (1.8)	4 (1.2)	2 (1.8)	<0.001
Urinary Diversion Orthotopic neobladder Ileal Conduit	/	88 (76) 28 (24)	/	/

**Table 2:** Baseline features after propensity score match analysis.

Variables N (%) Mean (SD)	Overall (170)	Bladder Cancer (85)	Healthy Population (85)	*p* Value
Gender				0.61
Male	122 (72)	59 (69)	63 (74)	
Female	48 (28)	26 (31)	22 (26)	
Age (yrs)	64 (11.4)	64 (9.8)	63 (12.9)	0.85
BMI (kg/m^2^)	26 (4.4)	26 (3.7)	26 (5.0)	0.52
Smoking history				0.20
Current	44 (26)	27 (32)	17 (20)	
Former	76 (45)	36 (42)	40 (47)	
Never	50 (29)	22 (26)	28 (33)	
Diabetes	21 (12)	10 (12)	11 (13)	1
Hypertension	84 (49)	39 (46)	45 (53)	0.44
Myocardial infarction history	25 (15)	9 (11)	16 (19)	0.19
Charlson Comorbidity Index	4 (1.6)	4 (1.3)	4 (1.9)	0.81
Urinary Diversion Orthotopic neobladder Ileal Conduit	/	63 (74) 22 (26)	/	/

**Table 3:** Comparison of HRQoL outcomes between Bladder Cancer population and Healthy population, before and after propensity score match analysis.

	Overall Cohort		*p* Value	PSM Cohort		***p*** **Value**
	Bladder Cancer (116)	Healthy Population (302)		Bladder Cancer (85)	Healthy Population (85)	
Global health status/QoL	69.8 ± 22.9	76.1 ± 17.7	0.003	69.4 ± 22.8	72.3 ± 19.3	0.38
Functional scales						
Physical functioning	90.9 ± 15.6	94.9 ± 11.7	0.005	91.8 ± 14.4	92.6 ± 14.2	0.73
Role functioning	88.5 ± 20.7	94.5 ± 14.3	0.001	89.4 ± 19.2	92.7 ± 15.7	0.22
Emotional functioning	79.6 ± 21.5	87.4 ± 16.7	<0.001	80.5 ± 22.3	87.9 ± 14.4	0.011
Cognitive functioning	92.0 ± 15.3	89.7 ± 15.1	0.17	92.9 ± 13.9	88.0 ± 15.0	0.028
Social functioning	86.9 ± 19.8	94.3 ± 13.7	<0.001	87.8 ± 19.3	92.2 ± 14.2	0.09
Symptoms scale						
Fatigue	18.2 ± 19.7	14.8 ± 17.2	0.09	18.1 ± 19.7	16.1 ± 17.1	0.48
Nausea and vomiting	3.7 ± 11.2	1.4 ± 5.2	0.005	3.1 ± 10.1	1.4 ± 5.3	0.16
Pain	12.6 ± 21.7	8.5 ± 16.2	0.037	11.6 ± 21.8	10.0 ± 16.1	0.60
Dyspnea	8.6 ± 15.9	6.7 ± 15.0	0.26	8.2 ± 16.2	9.4 ± 15.1	0.63
Insomnia	17.0 ± 26.6	13.4 ± 20.1	0.14	15.3 ± 26.0	17.6 ± 20.3	0.51
Appetite loss	8.3 ± 18.6	2.0 ± 8.4	<0.001	8.2 ± 19.2	2.4 ± 8.6	0.011
Constipation	13.2 ± 22.4	11.5 ± 21.1	0.46	12.9 ± 23.1	16.9 ± 24.5	0.28
Diarrhea	3.2 ± 11.6	3.9 ± 12.3	0.60	2.0 ± 9.4	4.7 ± 13.8	0.13
Financial difficulties	13.5 ± 22.8	3.3 ± 10.7	<0.001	11.8 ± 22.2	5.1 ± 13.1	0.018

## Data Availability

Deidentified database, study protocol, statistical analysis plan, and informed consent form will be made available “with publication”.

## References

[1] Kretschmer A., Grimm T., Buchner A., Jokisch F., Ziegelmüller B., Casuscelli J., Schulz G., Stief C.G., Karl A. (2020). Midterm Health-related Quality of Life After Radical Cystectomy: A Propensity Score–matched Analysis. Eur. Urol. Focus.

[2] Grobet-Jeandin E., Pinar U., Parra J., Rouprêt M., Seisen T. (2023). Health-related quality of life after curative treatment for muscle-invasive bladder cancer. Nat. Rev. Urol..

[3] Haraldstad K., Wahl A., Andenæs R., Andersen J.R., Andersen M.H., Beisland E., Borge C.R., Engebretsen E., Eisemann M., Halvorsrud L. (2019). A systematic review of quality of life research in medicine and health sciences. Qual. Life Res..

[4] Mastroianni R., Brassetti A., Krajewsky W., Zdrojowy R., Salhi Y.A., Anceschi U., Bove A.M., Carbone A., De Nunzio C., Fuschi A. (2020). Assessing the Impact of the Absence of Detrusor Muscle in Ta Low-grade Urothelial Carcinoma of the Bladder on Recurrence-free Survival. Eur. Urol. Focus.

[5] (2022). Muscle-Invasive and Metastatic Bladder Cancer.

[6] Nix J., Smith A., Kurpad R., Nielsen M.E., Wallen E.M., Pruthi R.S. (2010). Prospective Randomized Controlled Trial of Robotic versus Open Radical Cystectomy for Bladder Cancer: Perioperative and Pathologic Results. Eur. Urol..

[7] Bochner B.H., Dalbagni G., Sjoberg D.D., Silberstein J., Keren Paz G.E., Donat S.M.H., Coleman J.A., Mathew S., Vickers A., Schnorr G.C. (2015). Comparing open radical cystectomy and robot-assisted laparoscopic radical cystectomy: A randomized clinical trial. Eur. Urol..

[8] Khan M.S., Gan C., Ahmed K., Ismail A.F., Watkins J., Summers J.A., Peacock J.L., Rimington P., Dasgupta P. (2016). A single-centre early phase randomised controlled three-arm trial of open, Robotic, and Laparoscopic Radical Cystectomy (CORAL). Eur. Urol..

[9] Parekh D.J., Reis I.M., Castle E.P., Gonzalgo M.L., Woods M.E., Svatek R.S., Weizer A.Z., Konety B.R., Tollefson M., Krupski T.L. (2018). Robot-assisted radical cystectomy versus open radical cystectomy in patients with bladder cancer (RAZOR): An open-label, randomised, phase 3, non-inferiority trial. Lancet.

[10] Mastroianni R., Ferriero M., Tuderti G., Anceschi U., Bove A.M., Brassetti A., Misuraca L., Zampa A., Torregiani G., Ghiani E. (2022). Open Radical Cystectomy versus Robot-Assisted Radical Cystectomy with Intracorporeal Urinary Diversion: Early Outcomes of a Single-Center Randomized Controlled Trial. J. Urol..

[11] Messer J.C., Punnen S., Fitzgerald J., Svatek R., Parekh D.J. (2014). Health-related quality of life from a prospective randomised clinical trial of robot-assisted laparoscopic vs open radical cystectomy. BJU Int..

[12] Westhofen T., Eismann L., Buchner A., Schlenker B., Giessen-Jung C., Becker A., Stief C.G., Kretschmer A. (2022). Baseline Health-related Quality of Life Predicts Bladder Cancer-specific Survival Following Radical Cystectomy. Eur. Urol. Focus.

[13] Aaronson N.K., Ahmedzai S., Bergman B., Bullinger M., Cull A., Duez N.J., Filiberti A., Flechtner H., Fleishman S.B., de Haes J.C. (1993). The European organization for research and treatment of cancer QLQ-C30: A quality-of-life instrument for use in international clinical trials in oncology. J. Natl. Cancer Inst..

[14] Fayers P., Aaronson N., Bjordal K. (2001). EORTC QLQ-C30 Scoring Manual.

[15] Razdan S., Sljivich M., Pfail J., Wiklund P.K., Sfakianos J.P., Waingankar N. (2021). Predicting morbidity and mortality after radical cystectomy using risk calculators: A comprehensive review of the literature. Urol. Oncol..

[16] Hoeh B., Flammia R.S., Hohenhorst L., Sorce G., Chierigo F., Panunzio A., Tian Z., Saad F., Gallucci M., Briganti A. (2022). Outcomes of robotic-assisted versus open radical cystectomy in a large-scale, contemporary cohort of bladder cancer patients. J. Surg. Oncol..

[17] Yuh B., Wilson T., Bochner B., Chan K., Palou J., Stenzl A., Montorsi F., Thalmann G., Guru K., Catto J.W.F. (2015). Systematic review and cumulative analysis of oncologic and functional outcomes after robot-assisted radical cystectomy. Eur. Urol..

[18] Kowalewski K.F., Wieland V.L.S., Kriegmair M.C., Uysal D., Sicker T., Stolzenburg J.U., Michel M.-S., Haney C.M. (2023). Robotic-assisted Versus Laparoscopic Versus Open Radical Cystectomy—A Systematic Review and Network Meta-analysis of Randomized Controlled Trials. Eur. Urol. Focus.

[19] Clements M.B., Atkinson T.M., Dalbagni G.M., Li Y., Vickers A.J., Herr H.W., Donat S.M., Sandhu J.S., Sjoberg D.S., Tin A.L. (2021). Health-related Quality of Life for Patients Undergoing Radical Cystectomy: Results of a Large Prospective Cohort. Eur. Urol..

[20] Wilson T.G., Guru K., Rosen R.C., Wiklund P., Annerstedt M., Bochner B.H., Chan K.G., Montorsi F., Mottrie A., Murphy D. (2015). Best practices in robot-assisted radical cystectomy and urinary reconstruction: Recommendations of the Pasadena Consensus Panel. Eur. Urol..

[21] Desai M.M., Gill I.S. (2015). The devil is in the details: Randomized trial of robotic versus open radical cystectomy. Eur. Urol..

[22] Hoeh B., Flammia R.S., Hohenhorst L., Sorce G., Chierigo F., Panunzio A., Tian Z., Saad F., Gallucci M., Briganti A. (2023). Regional differences in total hospital costs for radical cystectomy in the United States. Surg. Oncol..

[23] Moschini M., Zamboni S., Soria F., Mathieu R., Xylinas E., Tan W.S., Kelly J.D., Simone G., Meraney A., Krishna S. (2019). Open Versus Robotic Cystectomy: A Propensity Score Matched Analysis Comparing Survival Outcomes. J. Clin. Med..

[24] Mastroianni R., Tuderti G., Ferriero M., Anceschi U., Bove A.M., Brassetti A., Misuraca L., Zampa A., Torregiani G., Covotta M. (2023). Open vs robotic intracorporeal Padua ileal bladder: Functional outcomes of a single-centre RCT. World J. Urol..

[25] Winters B.R., Wright J.L., Holt S.K., Dash A., Gore J.L., Schade G.R. (2018). Health Related Quality of Life Following Radical Cystectomy: Comparative Analysis from the Medicare Health Outcomes Survey. J. Urol..

[26] Catto J.W.F., Downing A., Mason S., Wright P., Absolom K., Bottomley S., Hounsome L., Hussain S., Varughese M., Raw C. (2021). Quality of Life After Bladder Cancer: A Cross-sectional Survey of Patient-reported Outcomes. Eur. Urol..

[27] Zimmermann K., Mostafaei H., Heidenreich A., Schmelz H.U., Shariat S.F., Mori K. (2021). Health-related quality of life in bladder cancer patients: Bladder cancer-specific instruments and domains. Part 2. Curr. Opin. Urol..

[28] Mastroianni R., Tuderti G., Ferriero M., Anceschi U., Bove A.M., Brassetti A., Misuraca L., D’Annunzio S., Guaglianone S., Gallucci M. (2023). Open versus robot-assisted radical cystectomy: Pentafecta and trifecta achievement comparison from a randomised controlled trial. BJU Int..

[29] Khetrapal P., Wong J.K.L., Tan W.P., Rupasinghe T., Tan W.S., Williams S.B., Boorjian S.A., Wijburg C., Parekh D.J., Wiklund P. (2023). Robot-assisted Radical Cystectomy Versus Open Radical Cystectomy: A Systematic Review and Meta-analysis of Perioperative, Oncological, and Quality of Life Outcomes Using Randomized Controlled Trials. Eur. Urol..

